# Actinomycosis in Relation to Meat Inspection

**Published:** 1892-06

**Authors:** W. L. Williams


### ACTINOMYCOSIS IN RELATION TO MEAT INSPECTION.

By W. L. Williams, V. S.

The now celebrated actinomycosis trial at Peoria, Ill., has led to a number of unusually interesting contributions upon this comparatively recently discovered disease, and as is usually the case in matters of so great importance and where such divergence of opinion exists, the discussion has at times become somewhat personal.

At an early stage of the discussion a somewhat caustic criticism was begun upon the evidence of Dr. R. W. Hickman, which eventually so angered him that he has been led to contribute an article to the April Journal (p. 239) consisting almost exclusively of an outpouring of wrath against the writer.

Along with this, however, when taken in connection with some other contributions, Dr. H. has made the statements and admissions which the caustic criticisms were intended to bring forth, and consequently we have no desire to have the acrimonious debate continued; and it is regretted that our colleague, who we have ever regarded as an honorable gentleman and accomplished veterniarian, should have so far misinterpreted the aim of the original criticisms as to regard them so wholly personal.

We have no reply to offer to these personalities beyond assuring Dr. H. that he has been misinformed in one respect, at least as to the character of our evidence, when in the closing lines of his recent contribution (Journal, p. 241) he states that we were put to the necessity by his expert testimony of hunting up actinomyces during the trial, and making measurements of them before going on the witness stand.

A careful perusal of the cross-examination will convince Dr. H. that no difference how far this may have been applicable to some other witnesses, it was quite untrue in our case.

There is, however, abundant material for a good-natured, serious discussion in Dr. Hickman's evidence and contributions. The real differences of opinion between those who favor condemnation of the carcasses of actinomycotic animals and those who do not, have in general been much magnified, and many have failed to heed the essential difference between the condemnation and destruction of meat.

Few, if any veterinarians or others, dispute the proposition that a purely local actinomycotic affection, when exercised, leaves the carcass healthy, in so far as actinomycosis is concerned ; but it is at the same time as universally admitted that the cansation of the disease is a microörganism, and only becomes apparent in an ordinary examination after the accumulation or growth of a sufficient amount of the fungus to lead to suppuration of the part, so that the disease is discovered rather by its results than its presence.

Those holding the views of Dr. Hickman generally admit that the presence of an external actinomycotic tumor on an otherwise apparently healthy animal, properly subjects the carcass of the animal to suspicion, and so the live animal is rejected for export to foreign nations and held for post mortem examination.

The divergence of opinion between those holding the different views begins in the main at the point where it is attempted to purge the carcass of the actinomycotic animal from suspicion.

An important preliminary question here arises. Shall the seller be compelled to prove the meat sound, or must the weight of evidence rest with the consumer and the meat passed unless proven unsound? Or once pronounced suspicious, shall it continue suspicious until proven sound? There is a wide range of opinion upon this subject, and we have already taken the stand that an animal once suspected on account of the presence of actinomycosis should be held as suspicious until proven sound beyond any reasonable doubt.

This would offer a more ready solution were we all thoroughly agreed as to the mode of invasion in this malady.

All admit that actinomycosis spreads by continuity and contiguity of tissues. If we all believed these to be the only methods, then the inspection of these suspected carcases would be greatly simplified, but we do not all so agree.

Dr. Hickman asserts, both in his evidence in the Peoria trial and in his contributions, that the smallest known virile organism of the actinomyces is sufficiently large to prevent its passage through the blood capillaries, and hence we would infer that to his thinking the only means for spreading are those offered by continuity or contiguity of tissue.

In spite, however, of these theoretical deductions, we find the disease in parts of the body where its presence cannot be traced to invasion from continuous or contiguous tissue, and where, in the present state of our anatomo-physiological knowledge, we can only account for its appearance by admitting that some form of the microörganism has passed safely through the blood capillaries, as, for example in the pericardium and spleen. The germ must also gain a small-sized venous capillary before it can be lodged on the surface of the liver just beneath Glissons capsule.

Equally positive and far more voluminous is the clinical evidence of a high order, that actinomycosis is disseminated over the body, through the agency of the lymphatic system, in spite of the fact that the lymph capillaries are usually considered as being even smaller than the blood capillaries, through which Dr. Hickman believes the germs cannot pass.

Dr. Hickman and those holding similar views, by condemning the meat of animals showing considerable actinomycotic lesions of the viscera, admit, moreover, that there exist other means of invasion than that by continuity and contiguity of tissue, since, were only these means of extension practicable, the affected parts could be readily and safely excised and the carcass freed from suspicion.

When we see actinomycosis beginning at the periphery of the body; say at the margin of the hip, at or near the feet, or in the prepuce, and then note the gradual centripetal dissemination of the disease, attacking seriatim, the lymphatic glands occurring in the road to the heart, *without affecting the intervening lymph channels themselves*, it seems quite clear that this is not invasion by continuity or contiguity, but dissemination through the agency of the lymph current.

It is to be admitted that the regular suppuration of these glands indicate clearly that, in a great measure, the disease germs are arrested in their lymphatic capillaries, and perhaps in some cases wholly destroyed by the suppurative process, but this admission does not invalidate the oft repeated clinical fact that, as a rule, enough germs pass through the lymph capillaries of a given gland to set up the same pathological processes in the next in the course of the lymph current, until finally the last gland in the course is affected, with the same opportunity for the passage of the actinomyces beyond it, as in any prior, more peripherally situated gland.

When, therefore, in cases of intestinal invasion we find small actinomycomata in the lymph glands in the intestinal walls, larger tumors in the mesenteric glands, and still larger and more notable in the lymphatic flexus about Pecquet's cistern ; and all this without a trace of disease in the lymph channels themselves, we are fully warranted in holding, that in all probability some of the germs have safely passed consecutively through the entire series of lymphatic glands from the intestine, and the last active sentinel having been evaded, reach the thoracic duct—an open gateway to the circulatory system of the right side of the heart. The heart once gained, the actinomyces are necessarily now forced into the lungs, where, doubtless, their course is very largely arrested by the pulmonary capillaries, and may proceed there to produce their characteristic pathological lesions.

In this respect actinomycosis bears a notable resemblance to tuberculosis and glanders, since the bacteria of both, regardless of the point of invasion, possess a marked tendency to become arrested in the pulmonary capillaries and establish their most typical lesions.

May it not be that many cases of actinomycosis in man and animals attributed to inhalation of the germ, are really due to the lodgment in the pulmonary capillaries of actinomyces derived from some distant part of the body, and carried by the circulatory system (blood and lymph) to the lungs?

Since it must be admitted that, in order to consistently condemn the carcass of an actinomycotic animal, the disease is capable of dissemination, either by the blood, or blood and lymph currents combined, we should examine carefully the comparative danger of meat infection in actinomycosis affecting the viscera, and the head or other external parts.

Dr. Hickman and his friends reject as dangerous those carcasses the viscera of which show evident lesions of actinomycosis, while they view as local and benign far more extensive lesions of the head. If, as Dr. H. claims, the germ were too small to pass through the blood capillaries, then, evidently, in so far as the blood is concerned, it would make no difference whatever as to the location of the actinomycotic lesions, so long as we confine our consideration to the danger from the living actinomyces themselves.

On the other hand, if we regard the lymphatic vessels as the chief or sole factor in disseminating the disease germs, we are confronted with some facts which are quite contradictory to that mode of procedure which condemns the animal with actinomycotic viscera and passes that with peripheral lesions.

If the actinomyces pass through lymph vessels and glands from the intestines, and reach the thoracic duct, they must pass thence into the vena cava through the heart, and into the pulmonary artery, reaching finally the pulmonary capillaries, where, if the germ be too large to pass, they must become lodged, and can only emerge from the the lungs by being once more taken up by the lymph vessels, in which case they would again return to the vena cava, heart, and lungs, and thus pursue an endless cycle, without a possibility of emerging from the internal viscera, so that if they were rejected, the rest of the carcass could not, according to our understanding of Dr. Hickman's views as to the impossibility of blood infection, be consistently condemned. On the other hand, in those cases showing lesions about the head or extremities, these parts being also abundantly supplied with lymphatics, it follows that actinomyces gaining admission to the lymph current, are carried along a vessel whose course is beset with the same lymphatic glands as are seen in the viscera, and unlike them, they lie deeply hidden in many cases in the interstices between muscles, where moderately affected glands are not discoverable by ordinary inspection, and are thus likely to be overlooked, and the surrounding tissues, including the actinomycotic gland, sold as healthful meat.

While disagreeing with Dr. Hickman as to the impossibility of dissemination through the agency of the blood circulation, we believe the lymphatic system to be the chief factor, and holding this view, our present anatomo-physiological knowledge certainly warrants the conclusion that, in so far as the danger to be feared from the ingestion of living actinomyces themselves is concerned, that the meat from animals suffering from actinomycosis of the head or extremities is immeasurably more to be feared than a like affection of the internal viscera.

